# Successive exposure to moderate hypoxia does not affect glucose metabolism and substrate oxidation in young healthy men

**DOI:** 10.1186/2193-1801-3-370

**Published:** 2014-07-21

**Authors:** Takuma Morishima, Kazushige Goto

**Affiliations:** Graduate School of Sport and Health Science, Ritsumeikan University, Kusatsu, Shiga Japan; Faculty of Sport and Health Science, Ritsumeikan University, 1-1-1, Nojihigashi, Kusatsu, Shiga 525-8577 Japan

**Keywords:** Moderate hypoxia, Postprandial glucose response, Substrate oxidation, Multiple meals, Healthy subjects

## Abstract

**Introduction:**

Exposure to hypoxia has been suggested to acutely alter glucose regulation. However, the effects of successive exposure to moderate hypoxia on postprandial glucose regulation and substrate oxidation pattern after multiple meals have not been elucidated.

**Purpose:**

We examined the effects of successive exposure to moderate hypoxia on metabolic responses and substrate oxidation pattern.

**Methods:**

Eight healthy men (21.0 ± 0.6 yrs, 173 ± 2.3 cm, 70.6 ± 5.0 kg, 23.4 ± 1.1 kg/m^2^) completed two experimental trials on separate days: a rest trial under normoxic conditions (FiO_2_ = 20.9%) and a rest trial under hypoxic conditions (FiO_2_ = 15.0%). Experimental trials were performed over 7 h in an environmental chamber. Blood and respiratory gas samples were collected over 7 h. Standard meals were provided 1 h (745 kcal) and 4 h (731 kcal) after entering the chamber.

**Results:**

Although each meal significantly increased blood glucose and serum insulin concentrations (*P* < 0.05), these responses did not differ significantly between the trials. There were no significant differences in areas under the curves for glucose or insulin concentrations over 7 h between the trials. No significant differences were observed in blood lactate, serum cortisol, free fatty acid, or glycerol concentrations over 7 h between the trials. The oxygen consumption ( ) and carbon dioxide production ( ) 3 h after entering the chamber were significantly higher in the hypoxic trial than in the normoxic trial (*P* < 0.05). However, the differences did not affect respiratory exchange ratio (RER). The average values of , , and RER did not differ between the trials.

**Conclusion:**

Seven hours of moderate hypoxia did not alter postprandial glucose responses or substrate oxidation in young healthy men.

## Introduction

There is an increasing body of evidence indicating the health benefits of exposure to hypoxia or exercise under hypoxic conditions. Rest or exercise under hypoxic conditions decrease body fat mass (Wiesner et al. 2010), blood pressure (Schobersberger et al. 2003), and arterial stiffness (Vedam et al. 2009; Nishiwaki et al. 2011) in various populations. Although several effects on metabolic and cardiovascular parameters have been proposed, the beneficial effects of hypoxic stimulation on glucose metabolism are especially attractive. People who live at high altitudes have lower blood glucose levels and lower risk of type 2 diabetes compared to those living at sea level (Picon Reategui 1963; Zubiate 2001; Castillo et al. 2007). In addition, lower values in the homeostatic model assessment (HOMA) have been found in people living at high altitudes, suggesting that high-altitude populations have higher insulin sensitivity (Baracco et al. 2006; Lindgarde et al. 2004). Although the mechanism underlying the glucose-lowering effect of sustained stays at high altitude is not fully understood, specific metabolic responses under hypoxic conditions may be involved because hypoxia itself stimulates glucose transporter (GLUT) 4-mediated glucose transport via several signaling pathways (Constable et al. 1988; Ploug et al. 1987; Cartee et al. 1991; Youn et al. 1994). Hypoxia promotes glucose uptake via an insulin-independent pathway (Youn et al. 1991, 1994) that stimulates GLUT 4 translocation to the plasma membrane.

Improvements in glucose metabolism via hypoxia have also been observed in data from laboratory-based experiments. Kelly et al. ( 2010) reported that plasma glucose response to a 75 g glucose load was significantly attenuated under severely hypoxic conditions (a simulated altitude of 4300 m) in healthy adults. Thus, severe hypoxic stimulation (a simulated altitude of > 4000 m) has a preventive effect on postprandial hyperglycemia. However, as the use of severe hypoxia would not be appropriate due to the risk of acute mountain sickness (e.g., headache, nausea, and anorexia), exposure to moderate hypoxia (a simulated altitude of < 3000 m) is more practical in terms of application for patients with impaired glucose tolerance. Mackenzie et al. ( 2011) demonstrated that blood glucose concentration was reduced after 1 h of exposure to moderate hypoxia (FiO_2_ = 14.6%). However, they only examined fasting glucose response after a short duration (1 h) of moderate hypoxia, and postprandial glucose responses over a day have not been elucidated when multiple meals are consumed.

The present study was performed to examine the effects of exposure to 7 h of moderate hypoxia on postprandial metabolic responses. We hypothesized that moderate hypoxia would attenuate postprandial blood glucose responses over a day.

## Methods

### Subjects

Eight healthy men (21.0 ± 0.6 yrs, 173 ± 2.3 cm, 70.6 ± 5.0 kg, 23.4 ± 1.1 kg/m^2^) participated in this study. The subjects were not participating in any training programs at the start of the study. All of the subjects were informed about the purpose of the study and experimental procedures, and written informed consent was obtained. The study was approved by the Ethics Committee for Human Experiments at Ritsumeikan University, Japan.

### Experimental design

The two experimental trials were carried out in a randomized crossover design. Each trial was separated by at least 7 days. The experimental trials consisted of two different measurements as follows: a rest trial under normoxic conditions (FiO_2_ = 20.9%) and a rest trial under hypoxic conditions (FiO_2_ = 15.0%). All trials were completed in an environmental chamber in which the temperature and relative humidity were maintained at 24°C and 40%, respectively.

**Figure 1:**
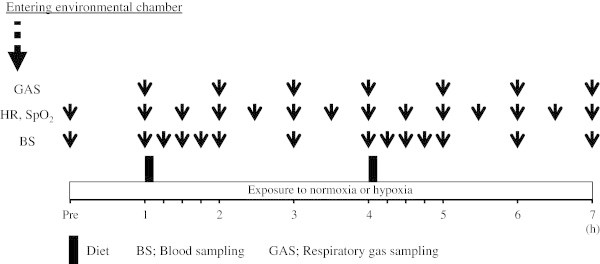
**Overview of the study design.**

The experimental protocol is shown in Figure 1. The two experimental trials were performed over 7 h following an overnight fast (at least 10 h). Throughout the experimental trials, subjects rested on a chair reading books or watching DVDs. Standard meals were provided 1 h and 4 h after entering the environmental chamber, and subjects were instructed to consume the meals within 7 min. The first meal consisted of 68.4% carbohydrate, 10.1% protein, and 21.5% fat (745 kcal). The second meals consisted of 66.9% carbohydrate, 10.1% protein, and 23.0% fat (731 kcal).

### Measurements on experimental trial days

Following an overnight fast, the subjects visited the laboratory in the morning and rested before the first blood collection. After a 30 min rest, a polyethylene catheter was inserted into an antecubital vein and a baseline blood sample was collected. Subsequently, respiratory gas, heart rate (HR) (Accurex Plus; Polar Electro Oy, Kempele, Finland), and percutaneous oxygen saturation (SpO_2_) (Smart Pulse; Fukuda Denshi, Tokyo, Japan) were recorded. During experimental trials, blood samples were collected at baseline and at 1 h (immediately before first meal), 1.25 h, 1.5 h, 1.75 h, 2 h, 3 h, 4 h (immediately before the second meal), 4.25 h, 4.5 h, 4.75 h, 5 h, 6 h, and 7 h (14 points in total, Figure 1). Respiratory gases were collected and analyzed using an automatic gas analyzer (AE300S, Minato Medical Science Co., Ltd, Osaka, Japan) at every hour (7 points in total). Appropriate calibrations of the O_2_ and CO_2_ sensors and the volume transducer were performed using calibration gases and 2 L syringe immediately before baseline measurement. From respiratory gas samples, oxygen consumption ( ), carbon dioxide production ( ), and ventilatory volume ( ) were determined. All respiratory variables were averaged in each 3-min period. The respiratory exchange ratio (RER) was determined from the  and  measurements. HR and SpO_2_ were recorded every 30 min (14 points in total).

### Blood analysis

Blood glucose, lactate, serum insulin, free fatty acid (FFA), glycerol, and cortisol concentrations were measured using whole-blood or serum samples. Serum samples were obtained by centrifugation for 10 min, and were stored at –80°C until analysis. The blood glucose and lactate concentrations were measured immediately after blood collection. Blood glucose and lactate concentrations were determined using an automated glucose analyzer (Free Style; Nipro Corporation, Osaka, Japan) and lactate analyzer (Lactate Pro 2; Arkray Inc., Kyoto, Japan), respectively. The glucose concentrations were analyzed in duplicate. The intraclass coefficient for duplicate measurements was 0.99. Serum insulin and FFA concentrations were measured by chemiluminescent enzyme immune assays (Fujirebio Inc., Tokyo, Japan) at a clinical laboratory (SRL Inc., Tokyo, Japan). Serum glycerol concentrations were measured in duplicate by enzyme-linked immunosorbent assay (Cayman Chemical Company, Ann Arbor, MI). Serum cortisol concentrations were measured by radioimmunoassay (RIA) using commercially available kits (Immunotech, Marseille, France). The intraassay coefficients of variation (CVs) were 1.1% for serum insulin, 2.2% for serum FFA, 3.3% for serum glycerol, and 4.4% for serum cortisol measurements.

### Statistical analysis

Data are expressed as means ± SE. Two-way analysis of variance (ANOVA) with repeated measures was used to test the interaction (trial × time) and main effect (trial, time). When ANOVA revealed a significant interaction or main effect, the Tukey–Kramer test was performed for post hoc analysis to identify differences. In all analyses, *P* < 0.05 was taken to indicate significance.

## Results

### SpO_2_ and HR

**Figure 2:**
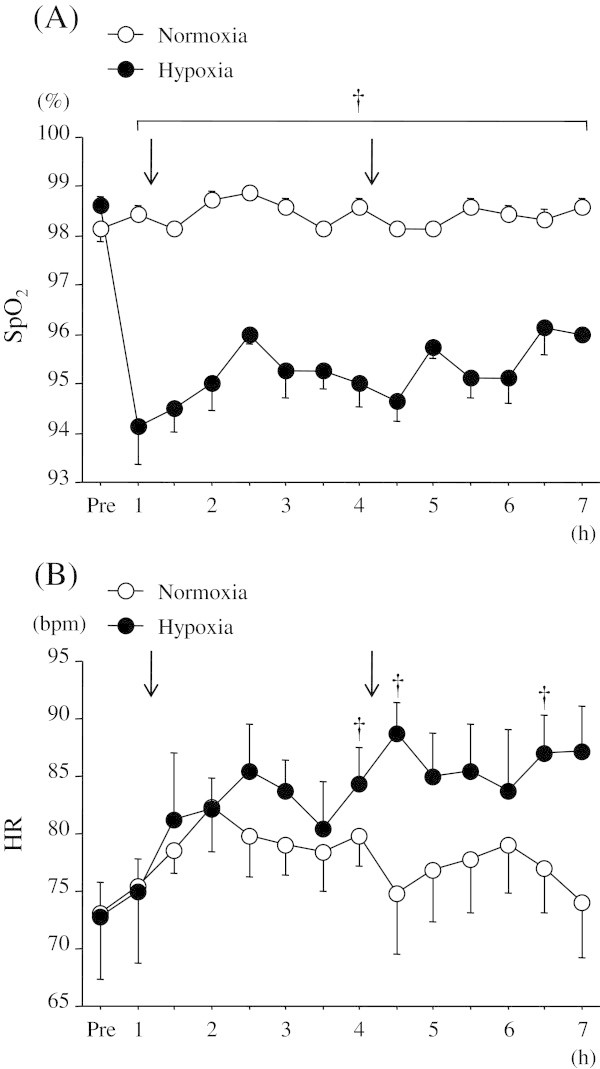
**Time courses of changes in SpO**_**2**_ **and HR over 7 h. (A)**; SpO_2_ and **(B)**; HR. The arrow indicates the time of meal consumption. ^†^ *P* < 0.05 vs. normoxia.

Figure 2 shows the time courses of changes in SpO_2_ and HR over 7 h. There were significant interaction (trial × time) and main effects for time and trial (*P* < 0.05) in SpO_2_. In the normoxic trial, SpO_2_ did not change at any time point over the 7 h experimental period. The hypoxic trials showed significantly lower values of SpO_2_ compared to the normoxic trial at all time points (*P* < 0.05). Average values of SpO_2_ during the 1–7 h after entering the chamber were 98 ± 1% for the normoxic trial and 95 ± 2% for the hypoxic trial (*P* < 0.05).

There were significant interaction (trial × time) and main effects for time and trial (*P* < 0.05) in HR. In the normoxic trial, HR did not change significantly over the 7 h experimental period. However, HR was significantly elevated at 2.5 h relative to the baseline value in the hypoxic trial (*P* < 0.05), and the value remained elevated throughout the rest of the measurement period. HR was significantly higher in the hypoxic trial than the normoxic trial at 4 h, 4.5 h, and 6.5 h (*P* < 0.05).

### Blood glucose and serum insulin responses

**Figure 3:**
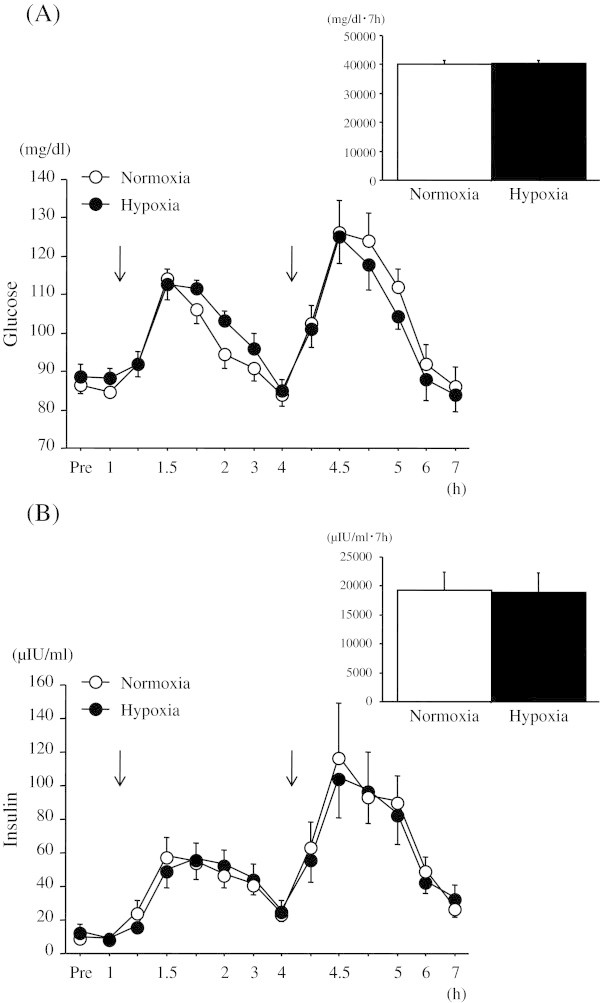
**Time courses of changes in blood glucose and serum insulin concentrations, and the area under the curve over 7 h. (A)**; blood glucose and **(B)**; serum insulin. The arrow indicates the time of meal consumption. ^†^ *P* < 0.05 vs. normoxia.

Figure 3 shows the time course of changes in the area under the curve (AUC) over 7 h for blood glucose and serum insulin concentrations. No significant interaction (trial × time) was found in blood glucose response. Although a significant main effect was observed for time (*P* < 0.05), there was no significant main effect of trial in blood glucose responses. When the time courses of changes in blood glucose concentrations over 7.5 h were compared, the AUC values did not differ significantly between the two trials.

Serum insulin response over 7 h was similar to the blood glucose response, and there was no significant interaction (trial × time). Although a significant main effect for time was found (*P* < 0.05), there was no significant main effect for trial. The AUC values for serum insulin concentrations over 7 h did not differ significantly between normoxic and hypoxic trials.

### Blood lactate, serum cortisol, FFA, and glycerol responses

**Figure 4:**
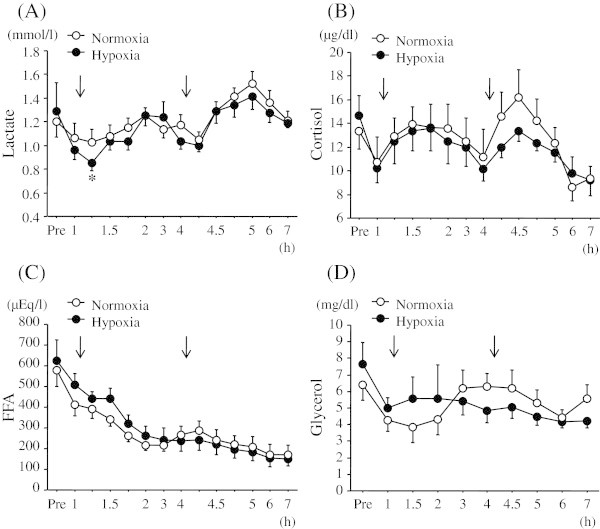
**Time courses of changes in blood lactate, serum cortisol, FFA, and glycerol concentrations over 7 h. (A)**; blood lactate, **(B)**; serum cortisol, **(C)**; serum FFA, and **(D)**; serum glycerol. The arrow indicates the time of meal consumption.

Figure 4 shows the time courses of changes in blood lactate, serum cortisol, FFA, and glycerol concentrations over 7 h. For blood lactate responses, there was no significant interaction (trial × time). Although a significant main effect for time was observed (*P* < 0.05), there was no significant main effect for trial in blood lactate responses. In the hypoxic trial, blood lactate concentration decreased significantly at 1.25 h relative to the baseline value (*P* < 0.05). At 5 h (at 1 h after second meal), blood lactate concentration increased significantly relative to the baseline value in the normoxic trial. There was no significant interaction (trial × time) or main effect for time or trial (*P* < 0.05) in serum cortisol concentration.

Serum FFA responses did not show a significant interaction (trial × time). Although a significant main effect for time was observed (*P* < 0.05), there was no significant main effect for trial in serum FFA responses.

There was no significant interaction (trial × time) or main effect for time or trial (*P* < 0.05) in serum glycerol concentration.

### Respiratory gas parameters

Table 1 shows the time courses of changes in respiratory gas parameters. No significant differences in baseline variables (1 h after entering the chamber) were observed between the trials. For  responses, there was no interaction (trial × time). However, significant main effects for time and trial were observed (*P* < 0.05).  was significantly elevated at 5 h and 7 h relative to the value at 1 h in the normoxic trial (*P* < 0.05) and at 3 h in the hypoxic trial (*P* < 0.05). At 3 h,  was significantly higher in the hypoxic trial compared to the normoxic trial (*P* < 0.05). However, average values of  over 7 h were not significantly different between the two trials.

**Table 1:** **Respitatory gas parameters**

		1 h	2 h	3 h	4 h	5 h	6 h	7 h	Average
(ml/min)	Normoxia	254 ± 10	268 ± 12	267 ± 15	266 ± 18	282 ± 13*	271 ± 11	294 ± 17*	272 ± 13
	Hypoxia	251 ± 15	278 ± 15*	291 ± 19*†	272 ± 15	285 ± 17*	291 ± 13*	272 ± 9	277 ± 14
(ml/min)	Normoxia	211 ± 10	222 ± 10	221 ± 12	224 ± 16	244 ± 14*	239 ± 10*	260 ± 12*	232 ± 11
	Hypoxia	198 ± 14	225 ± 15	241 ± 17*†	234 ± 15*	246 ± 16*	256 ± 11*†	245 ± 9	235 ± 13
(ml/min)	Normoxia	8.5 ± 0.4	7.9 ± 0.4	8.0 ± 0.5	8.6 ± 0.7	8.6 ± 0.6	9.0 ± 0.4	9.6 ± 0.4	8.6 ± 0.4
	Hypoxia	8.7 ± 0.5	9.1 ± 0.5*	10.0 ± 0.5*†	9.9 ± 0.6*†	10.4 ± 0.6*†	10.4 ± 0.4	10.1 ± 0.5*	9.8 ± 0.5†
RER	Normoxia	0.83 ± 0.02	0.82 ± 0.01	0.83 ± 0.01	0.84 ± 0.01	0.86 ± 0.02	0.88 ± 0.02	0.89 ± 0.03	0.85 ± 0.01
	Hypoxia	0.78 ± 0.02	0.81 ± 0.02	0.83 ± 0.02	0.86 ± 0.02*	0.85 ± 0.02*	0.88 ± 0.01*	0.89 ± 0.01*	0.85 ± 0.01

Mean ± SE. *P < 0.05 vs. 1 h. ^†^ *P* < 0.05 vs. Normoxia.

Although there was no significant interaction (trial × time) in , significant main effects for time and trial were observed (*P* < 0.05).  was significantly elevated at 5 h, 6 h, and 7 h relative to the value at 1 h in the normoxic trial, and at all time points in the hypoxic trial. At 3 h and 6 h,  was significantly higher in the hypoxic trial compared to the normoxic trial. However, average values of  over 7 h were not significantly different between the two trials.

Although there was no significant interaction (trial × time) in , significant main effects for time and trial were observed (*P* < 0.05). In the normoxic trial,  did not change significantly over the 7 h experimental period. However,  was significantly elevated at all time points relative to the value at 1 h in the hypoxic trial (*P* < 0.05). At 3–6 h,  was significantly higher in the hypoxic trial than in the normoxic trial (*P* < 0.05). Average values of  over 7 h were significantly higher in the hypoxic trials than in the normoxic trial (*P* < 0.05).

There was no significant interaction (trial × time) in RER. Although a significant main effect for time was observed (*P* < 0.05), there was no significant main effect for trial. Although RER did not change significantly over 7 h in the normoxic trial, significant increases relative to the value at 1 h were observed at 4 h, 5 h, 6 h, and 7 h in the hypoxic trial (*P* < 0.05). However, average values of RER over 7 h were not significantly different between the two trials.

## Discussion

We investigated the effects of 7 h of exposure to moderate hypoxia on metabolic responses to multiple meals. Our main finding was that postprandial glucose responses and substrate oxidation patterns were not significantly affected by 7 h of moderate hypoxia. In the present study, physiological effects of hypoxia were confirmed by comparisons of , , HR and SpO_2_ between hypoxic and normoxic trials. However, there were no differences in blood glucose, serum insulin, glycerol or FFA responses over 7 h. This suggests that exposure to moderate hypoxia does not exert any change in the blood glucose responses to mixed meals.

Exposure to hypoxia has been suggested to acutely alter glucose regulation. Mackenzie et al. ( 2011) reported that blood glucose concentration was significantly reduced after 1 h of moderate hypoxia. In contrast, in the present study, the time courses of changes in blood glucose concentration were not significantly affected by 1 h or 7 h of exposure to moderate hypoxic conditions. Differences in subject characteristics may explain these contradictory outcomes between the study of Mackenzie et al. ( 2011) and the present study. Although the previous study by Mackenzie et al. ( 2011) recruited patients with type 2 diabetes, the subjects in the present study were healthy adults with normal glycemic regulation. Patients with type 2 diabetes have lower glucose uptake ability by insulin (Gierach et al. 2014) and insulin-independent signaling pathways (Barnes et al. 2002; Sriwijitkamol et al. 2006) due to increased fat mass (Saltiel and Olefsky 1996) or impaired mitochondrial function (Khan et al. 2014). Nonetheless, sustained elevation of blood glucose concentration is thought to enable enhanced glucose uptake by hypoxic stimuli. In the case of healthy individuals with normal glycemic regulation, blood glucose regulation appears to be robust, at least under moderate hypoxic conditions. In addition, SpO_2_ levels of the present subjects in the hypoxic trial were modest (95 ± 2%) and may have been insufficient to augment glucose uptake. Chen et al. ( 2009) reported that glucose uptake in the heart was significantly promoted by moderate hypoxia when SpO_2_ levels were below 80%. Therefore, the influence of hypoxia on postprandial glycemic regulation is not conclusive, and further studies are necessary to determine whether moderate hypoxia over a day improves glycemic regulation in patients with type 2 diabetes. Moreover, as muscle contraction is a strong stimulus for glucose uptake (Holloszy 2003), the combined effects of hypoxia and exercise on postprandial glucose response should be investigated.

The  and  at 3 h after entering the chamber were significantly higher in the hypoxic trial than the normoxic trial. The elevated  and  could be associated with slightly higher values of VE in the hypoxic trial. It is well known that acute hypoxic exposure induces hyperventilation (Dempsey and Forster 1982; Easton et al. 1986), which may cause an overestimation of CO_2_ production (Ferrannini 1988). However, RER did not differ between the trials in the present study. In addition, there were no significant differences in serum FFA or glycerol concentrations between the trials, suggesting that the substrate oxidation pattern did not change with exposure to moderate hypoxia. Several previous studies have indicated that hypoxia increases carbohydrate oxidation (Brooks et al. 1991; Roberts et al. 1996a, [b]). Enhanced reliance on plasma glucose oxidation for energy production under hypoxic conditions leads to the promotion of carbohydrate oxidation during rest or exercise (Brooks et al. 1992; Peronnet et al. 2006). Although the present results do not support this suggestion, previous studies have indicated enhancement of carbohydrate oxidation by hypoxia in elite endurance athletes, with marked differences between endurance athletes and sedentary individuals (Havel et al. 1963, 1964; Hurley et al. 1986; Bircher and Knechtle 2004). Furthermore, most previous studies (Brooks et al. 1991, 1992; Roberts et al. 1996a, [b]; Peronnet et al. 2006) used severe hypoxia (altitude > 4000 m). Therefore, differences in the levels of hypoxia (i.e., altitude) between previous studies and the present study may explain the contradictory outcomes.

## Conclusion

7 h of moderate hypoxia did not alter postprandial glucose response or substrate oxidation in healthy young men. Although the present study was carried out in healthy individuals, further experiments in those with impaired glycemic regulation are required.

## Abbreviations

ANOVA: Analysis of variance
AUC: Area under the curve
FFA: Free fatty acid
GLUT: Glucose transporter
HOMA: Homeostasis model assessment
HR: Heart rate
RER: Respiratory exchange ratio
RIA: Radioimmunoassay
SpO_2_: Percutaneous oxygen saturation
VCO_2_: Carbon dioxide production
VE: Ventilatory volume
VO_2_: Oxygen consumption.
